# Case 4/2017 - Young Male Marathoner with Heart Failure Due to Dilated Cardiomyopathy

**DOI:** 10.5935/abc.20170124

**Published:** 2017-08

**Authors:** Hilda Sara Montero Ramirez, Rafael Amorim Belo Nunes, Vera Demarchi Aiello

**Affiliations:** Instituto do Coração (InCor) HC-FMUSP, São Paulo, SP - Brazil

**Keywords:** Heart Failure, Cardiomyopathy, Dilated, Physical Exertion, Athletes, Sports

The patient was a 22-year-old male marathoner, from the town of Ranchinho, Bahia state, Brazil, coming from the city of São Paulo, São Paulo state, Brazil, hospitalized due to cardiogenic shock following chest pain and syncope after physical exertion.

According to his father, the patient had convulsions during childhood and an episode of rheumatic fever during adolescence, having undergone antibiotic prophylaxis with benzathine penicillin for 2 years, which was discontinued spontaneously, without disease recurrence. In addition, he reported recent alcohol abuse and depressive symptoms, but neither his family nor friends knew any illicit drug use.

In the preceding year, the patient had sporadic episodes of dyspnea and chest discomfort on exertion. He maintained his training and running practices, although less intensely, because of lower limb pain and weakness in past months, until 5 days ago (Aug 26, 2009), when, right after a training session, he experienced sudden chest pain, cough with hemoptysis, general weakness, shivering, mental confusion and syncope. The patient sought a hospital close to his dwelling, being admitted for observation.

His ECG (Aug 27) showed supraventricular rhythm, 125 beats per minute, indirect signs of right atrial overload, left ventricular overload, and secondary changes of ventricular repolarization (Figure 1).

**Figure 1:**
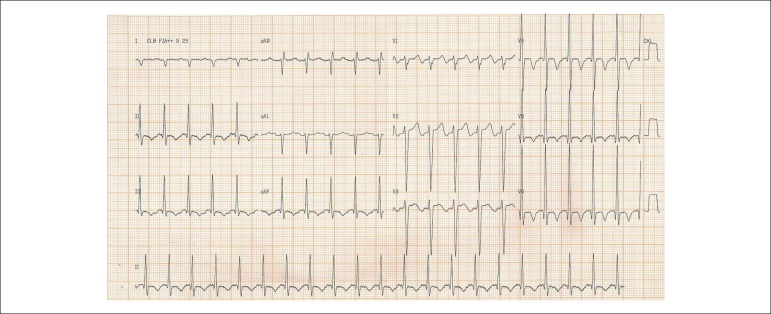
ECG (Aug 27): supraventricular tachycardia, axis shifted to the right, 125 beats per minute, indirect signs of right atrial overload, left ventricular overload, secondary changes of ventricular repolarization.

His enzyme measurements were as follows: CPK 120 IU/L, CK-MB 29 UI/L, and troponin 0.77 ng/L.

His echocardiogram (Aug 29) revealed the following: diameters of the aorta 28 mm, left atrium 42 mm, right ventricle 20 mm, and left ventricle (diastole/systole) 75/66 mm; ejection fraction 25% (Teicholz); and interventricular septum and posterior wall thickness of 9 mm and 10 mm, respectively. There was left ventricular diffuse hypokinesis, with restrictive filling, and moderate mitral and tricuspid regurgitation.

The patient became lethargic with tachypnea. His physical exam (Aug 30) showed heart rate of 115 beats per minute, respiratory rate of 30 breaths per minute, blood pressure of 92/54 mmHg, and O_2_ saturation of 90% with O_2_ catheter at a 3 L/min flow rate. His jugular venous pressure was elevated (++/4), and his pulmonary auscultation, normal. His cardiac auscultation revealed mitral and tricuspid heart murmurs (+++/6) at systole and no accessory heart sound. His abdomen was flaccid and painless, without visceromegaly. His lower limbs showed no edema. His pulses were symmetrical and thin, and the peripheral perfusion was poor.

Intravenous dopamine was initiated to treat shock, and enoxaparin and clopidogrel were added.

The patient experienced a new episode of chest pain and dyspnea, with lowering of the level of consciousness, requiring orotracheal intubation for ventilatory support. The patient developed shock refractory to volume and noradrenaline administration, being transferred to the Instituto do Coração (InCor).

His physical exam on admission at InCor (Aug 31) revealed poor general state, cold extremities, mechanical ventilation with endotracheal intubation, and inaudible blood pressure. His pulmonary auscultation evidenced diffuse rhonchi. His cardiac auscultation revealed a third heart sound and mitral heart murmur (+++/6) at systole. The abdomen showed no abnormality, and the extremities, no edema.

His laboratory tests (Aug 31) were as follows: hemoglobin, 17.4 g/dl; hematocrit, 59%; leukocytes, 20900/mm^3^ (4% band neutrophils, 76% segmented neutrophils, 11% lymphocytes, 9% monocytes); platelets, 288000/mm^3^; ferritin, 258 ng/mL; CK-MB, 12 ng/mL; troponin I, 4.11 ng/m L; CPK, 3168 U/L; AST, 145 U/L; ALT, 61 U/L; LDH, 387 U/L; C-reactive protein, 88.5 mg/L; urea, 45 mg/dL; creatinine, 3.1 mg/dL (GF = 27 mL/min/1.73 m^2^); sodium, 140 mEq/L; potassium, 6.4 mEq/L; arterial lactate, 15 mg/dL; TP (INR) 6.6; TTPA incoagulable; D dimer, 2460 ng/mL. His arterial blood gas analysis revealed: pH 7.15; paCO_2_ 60.2 mm Hg; paO_2_ 54.2 mm Hg; O_2_ saturation 79.6%; bicarbonate 20.2 mEq/L and base excess (-)10 mEq/L. His protein electrophoresis revealed: total protein, 4.9 g/dL; albumin, 2.3 g/dL; and globulins: alpha 1, 0.3 g/dL; alpha 2, 0.6 g/dL; beta, 0.7 g/dL; gamma, 1 g/dL. Serologies for Chagas disease, hepatitis C, and HIV were negative, and IgG antibodies were positive for hepatitis A and B, cytomegalovirus and toxoplasmosis.

His ECG (Aug 31) showed atrial fibrillation, 140 beats per minute, left ventricular overload, pointed T wakes in V_1_ and V_2_, and inverted in V_5_ and V_6_ (Figure 2).

**Figure 2:**
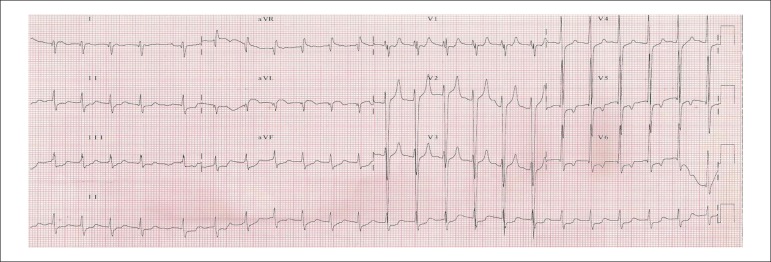
ECG (Aug 31): atrial fibrillation, axis shifted to the right, 140 beats per minute, left ventricular overload, pointed T waves in V_1_ and V_2_ and inverted in V_5_ and V_6_.

His new echocardiogram (Aug 31) evidenced: diameters of the aorta 27 mm, left atrium 45 mm, right ventricle 42 mm, left ventricle (diastole/systole) 74x68 mm; ejection fraction (Teicholz) 17%; and similar interventricular septum and posterior wall thickness, 9 mm. The ventricles were dilated and diffusely hypokinetic, and there was no valvular abnormality, but signs of pulmonary hypertension. His transesophageal echocardiogram (Sept 2) showed a pedunculated thrombus adhered to the left ventricular anterior wall, measuring 2.18 x 1.16 cm.

Intraaortic balloon for circulatory support was initiated on August 31.

The patient improved his hemodynamic findings, but fever appeared.

The intraaortic balloon was removed on September 4, when the hemodynamic measures were as follows: blood pressure 150/72 mm Hg; pulmonary artery pressure 34/15 mm Hg; central venous pressure, 11 mm Hg; cardiac index 3.72 L/min.m^2^; left ventricular systolic volume index 48 mL/m^2^/beat; systemic vascular resistance index 1741 dyn.s.m^2^.cm^-5^.

Antibiotic therapy with vancomycin, piperacillin and tazobactam was initiated.

His laboratory tests (Sept 4) revealed: hemoglobin, 10.4 g/dL; hematocrit, 33%; leukocytes, 10400/mm^3^ (75% neutrophils); platelets, 155000/mm^3^; TP (INR) 1.1; TTPA (rel) 1.25; PCR, 130 mg/L; CPK, 1124 U/L; urea, 23 mg/dL; creatinine, 0.6 mg/dL; sodium, 139 mEq/L; potassium, 3.2 mEq/L; AST, 98 U/L; and ALT, 62 U/L.

In the following days, his renal function (creatinine, 2.6 mg/dL) and pulmonary congestion worsened.

His state of consciousness oscillated from agitation to somnolence. His neurological assessment comprised skull tomography and cerebrospinal fluid analysis. The latter was as follows: colorless and clear; negative for bacteria and fungi; glucose, 70 mg/dL; chloride, 129 mEq/L; proteins, 38 mg/dL; lactate, 9.2 mg/dL; cells, zero and 1 red blood cell/mL (Sept 9).

His skull tomographies (Sept 8 and 10) revealed hypoattenuating areas in the white and gray matters of the right parietal and occipital regions, suggesting acute ischemic lesions.

His chest tomography (Sept 8) revealed significant cardiomegaly, moderate bilateral pleural effusion, atelectasis of the adjacent parenchyma and diffuse ground glass opacity, more evident in the lower lobes.

His abdominal tomography (Sept 8) showed hepatomegaly, homogeneous liver with blunt borders, dilatation of the inferior vena cava and suprahepatic veins; hyperattenuating gall bladder content, suggesting biliary mud. The spleen, pancreas, kidneys, adrenal glands and abdominal aorta showed no abnormality.

The new echocardiographic assessments (Sept 9 and 17) showed no change as compared to the initial one, except for a reduction in right ventricular dilatation and hypokinesis.

On the 11^th^ day of admission, the fever recurred and purulent sputum appeared. The latter improved with the addition of colistimethate to therapy. However, after three days, the patient had acute pulmonary edema with arterial hypertension, and persistent fever. Because of the presence of yeast cells with pseudohyphae in tracheal secretion, fluconazole was introduced.

The blood cultures collected (Sept 11) were positive for coagulase-negative *staphylococci* sensitive to teicoplanin, vancomycin and the sulfamethoxazole-trimethoprim association.

On the 20^th^ day of admission, the patient had polymorphic ventricular tachycardia and cardiopulmonary arrest, which reversed with electrical defibrillation with 200 J, but recurred short after, degenerating to ventricular fibrillation and pulseless electrical activity, which also reversed. The patient received intravenous amiodarone (300 mg), but developed shock and acute pulmonary edema.

His laboratory tests (Sept 19) were as follows: hemoglobin, 12.1 g/dL; hematocrit, 39%; leukocytes, 26900/mm^3^ (14% band neutrophils, 72% segmented neutrophils); platelets, 300000/mm^3^; TP (INR) 7.5; TTPA (rel) 1.92; sodium, 155 mEq/L; potassium, 5.8 mEq/L; magnesium, 1.9 mmol/L; urea, 89 mg/dL; creatinine, 3.9 mg/dL; lactate, 145 mg/dL. His arterial blood gas analysis was as follows: pH 7.42; paCO_2_ 35 mm Hg; paO_2_ 108 mm Hg; O_2_ saturation 98%; bicarbonate 22 mEq/L and base excess (-)1.5 mEq/L.

The patient remained in shock with fever, which did not improve with the intraaortic balloon and change from fluconazole to amphotericin and introduction of the sulfamethoxazole-trimethoprim association. He died in pulseless bradycardia on the 22^nd^ day of admission (Sept 21, 2009).

## Clinical aspects

The patient was a 22-year-old male marathoner with fatal heart failure for 1 year. The clinical data reported were dyspnea and precordial discomfort on exertion. The patient maintained his trainings and running practice less intensely due to lower limb pain and weakness in the last months, until he was hospitalized due to chest pain, dyspnea, hemoptysis, mental confusion and syncope. During hospitalization, his clinical findings rapidly and progressively deteriorated. His electrocardiogram on admission revealed signs of right atrial overload, left ventricular overload and secondary ventricular repolarization changes. The left ventricle was diffusely hypokinetic and the diastolic function pattern was restrictive. There was moderate mitral and tricuspid valve regurgitation. These findings suggest cardiomyopathy with important hemodynamic repercussion.

The causes of cardiomyopathy in young individuals are: idiopathic cardiomyopathy, infectious myocarditis and autoimmune myocarditis.^1^ Cardiotoxicity can also be observed in exposure to certain agents, such as alcohol, cocaine, heavy metals and antineoplastic drugs, such as anthracyclines and cyclophosphamide.

Idiopathic dilated cardiomyopathy is the most common cause of heart failure in young individuals, 30% to 50% of the cases being familial and associated with inherited genetic mutations.^2,3^ Some patients have heart failure of rapid progression and refractory to treatment, one of the most frequently found etiologies in heart transplant lists.^4^

Lymphocytic myocarditis can be triggered by different infectious agents, viruses being the most frequent ones. Lymphocytic myocarditis can progress with acute, subacute or chronic heart failure. More than 20 viruses, such as Coxsackievirus B, adenovirus, parvovirus B19, cytomegalovirus and human herpesvirus 6, have been related to the risk for myocarditis.^5^ Some groups, such as children and immunocompromised patients, are at higher risk to develop rapidly progressive or fulminant heart failure related to viral myocarditis. Other infectious agents, such as bacteria, rickettsia and fungi, are occasionally associated with myocarditis, but less commonly than viruses. In Brazil, where Chagas disease is endemic in some regions, some patients can have rapidly progressive myocardial impairment. Although our patient was born in an area potentially endemic for Chagas disease, his serology was negative.

Autoimmune myocarditis can lead to acute or subacute heart failure with rapid decompensation. Giant cell myocarditis is a rare disease, mediated by abnormalities in T lymphocyte function, affects mainly young and middle-aged individuals, has a rapid course and high mortality rate. The initial presentation in 75% of the cases is rapidly progressive heart failure, while the rest present with cardiac arrhythmias and findings similar to acute myocardial infarction. Around 20% of the patients with giant cell myocarditis have associated autoimmune conditions, such as inflammatory intestinal disease, thyroiditis, celiac disease, rheumatoid arthritis.^6^ Similarly to giant cell myocarditis, eosinophilic myocarditis can be characterized by rapidly progressive and potentially fatal heart failure, being occasionally related to exposure to drugs or exogenous agents. Cardiac sarcoidosis is also a differential diagnosis, although there was no report of previous impairment of organs, such the lungs and liver. Usually, patients with cardiac sarcoidosis have changes in the cardiac conduction system, with total atrioventricular block in up to 30% of the cases, related to the presence of granulomas and scars in the basal region of the interventricular septum.^2^

Acute myocardial infarction is a frequent cause of acute heart failure, is more common in middle-aged and old patients with atherosclerotic risk factors and/or established disease. In our case, the patient was young, had no classical risk factor for cardiovascular disease, and his clinical and laboratory findings did not suggest acute coronary syndrome.

Between the fifth and sixth days of hospitalization, the patient had a significant clinical deterioration, with respiratory and hemodynamic failure, change in his level of consciousness, requiring invasive ventilatory support and vasoactive drugs. His physical exam showed signs of acute heart failure with the third heart sound and pulmonary congestion, poor peripheral perfusion with elevated capillary filling time and increased central venous pressure, characterized by the presence of jugular venous distention. His laboratory tests on the occasion revealed several data of poor prognosis due to organic dysfunctions, such as elevation of nitrogen compounds, arterial lactate, liver enzymes, acidosis, hypoxemia, as well as increased levels of inflammatory markers, such as C-reactive protein, leukocytosis and D dimer. His electrocardiogram evidenced atrial fibrillation with high response, left ventricular overload and ventricular repolarization changes. The comparison of the new echocardiography with the previous one showed right ventricular dilatation and hypokinesis, and indirect signs of pulmonary hypertension. The transesophageal echocardiogram revealed a pedunculated thrombus on the left ventricular anterior wall, which increased his cardioembolic risk, being probably related to the presence of right frontal and parietal cerebral ischemic lesions later observed on skull tomography.

In some patients with acute or chronic heart failure, some conditions, such as underlying disease, infections, pulmonary thromboembolism (PTE), arrhythmias, myocardial ischemia and anemia, can trigger or worsen decompensation. Infection and PTE might have contributed to our patient's clinical deterioration. A recent study has reported a 17.8% PTE prevalence in individuals hospitalized due to syncope.^7^ In our patient, the new right ventricular dilatation and dysfunction associated with worsening of the respiratory findings, hypoxemia and shock might suggest PTE as the possible cause of decompensation. Viral and bacterial infections are among the most common causes of decompensation in patients hospitalized with heart failure.^4^ This higher incidence of infectious diseases in patients with heart failure is multifactorial, resulting from the interaction of different abnormalities, such as immunological changes in critically ill patients, nutritional deficiencies and higher need for invasive diagnostic and therapeutic procedures. Despite the treatment with large spectrum antibiotics because of fever and suggestive signs of sepsis, in addition to the hemodynamic support during hospitalization, the patient experienced hemodynamic instability and refractory shock. (**Hilda Sara Montero Ramirez, MD, and Rafael Amorim Belo Nunes, MD**)

Diagnostic hypotheses: congestive heart failure; myocarditis (autoimmune? viral?); cardioembolic ischemic stroke; nosocomial bronchopneumonia; cardiogenic and septic shock. (**Hilda Sara Montero Ramirez, MD, and Rafael Amorim Belo Nunes, MD**)

## Postmortem examination

**Figure 3:**
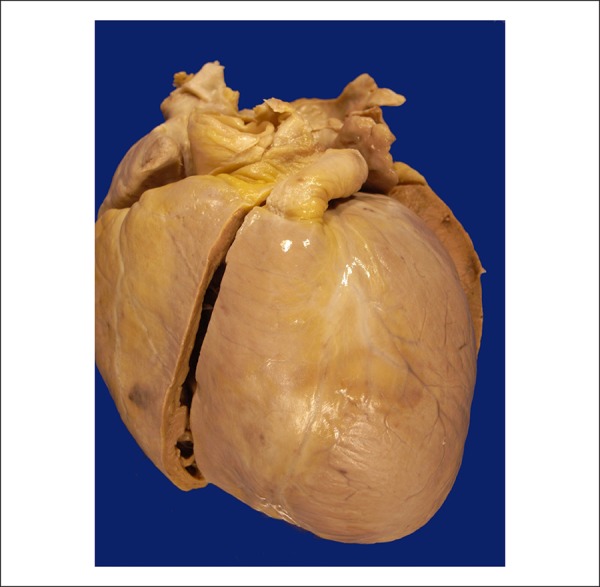
External view of the heart showing volume enlargement and globose shape, and bright epicardium.

**Figure 4:**
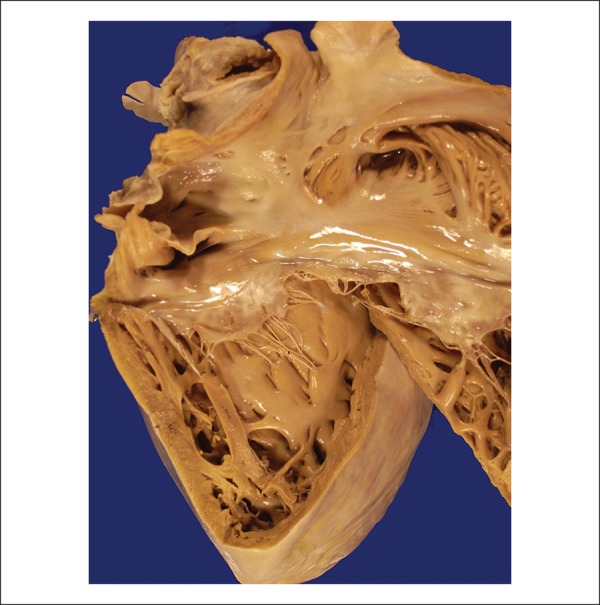
Opened right cardiac chambers showing atrial and ventricular dilatation, and mild myocardial hypertrophy.

**Figure 5:**
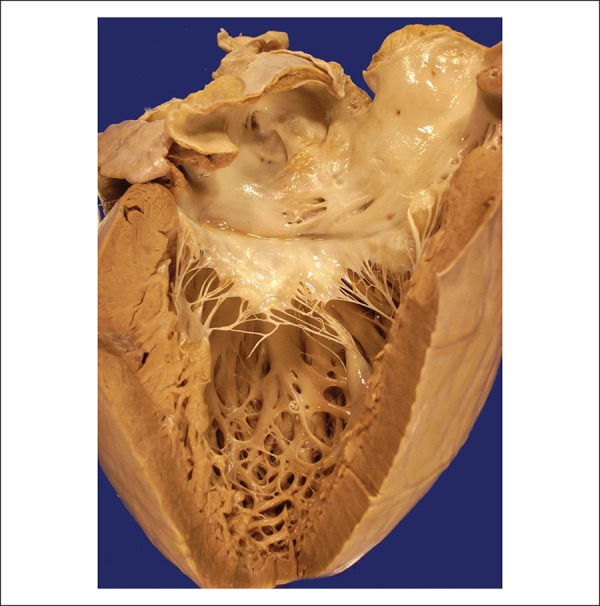
Opened left atrium and ventricle showing dilatation of both cardiac chambers and hypertrophic myocardium.

The heart weighed 590 g (normal, up to 350 g), was moderately enlarged and had a globose shape (Figure 3). The epicardial surface had small pericoronary fibrous thickenings. When opened, dilatation of all chambers with mild hypertrophy of the ventricular and atrial walls was observed (Figures 4 and 5). Neither the atrioventricular nor the arterial valves had abnormalities. There were neither cavitary thrombi nor endocardial thickening. The coronary arteries had usual origins and showed no significant obstructive lesion.

**Figure 6:**
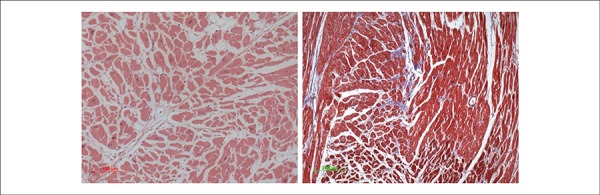
Photomicrographs of the myocardium showing hypertrophy of cardiomyocytes and interstitial fibrosis (blue areas in the right panel). Left panel: Hematoxylin- Eosin, 20X; right panel: Masson’s trichrome, 10X.

**Figure 7:**
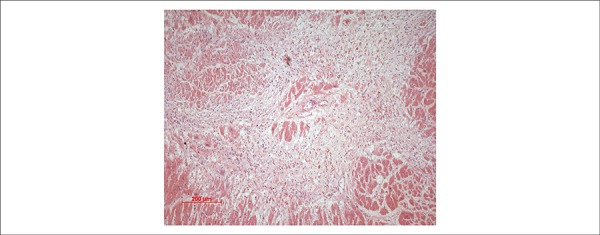
Photomicrograph of an area of organizing myocardial microinfarct. Hematoxylin-Eosin, 10X.

**Figure 8:**
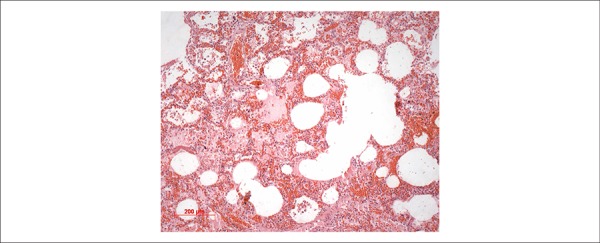
Photomicrograph of the lungs showing diffuse alveolar hemorrhage. Hematoxylin-Eosin, 10X.

The microscopic exam showed moderate to marked hypertrophy of cardiomyocytes, with focal myocardial interstitial fibrosis (Figure 6) and areas of organizing microinfarcts (Figure 7). There were signs of terminal shock, such as centrilobular liver necrosis, renal acute tubular necrosis and recent pulmonary alveolar hemorrhage (Figure 8), in addition to signs of systemic embolism, such as recent infarcts in the brain (right parietal and occipital) and spleen. (**Prof. Vera Demarchi Aiello, MD**)

**Diagnosis:** Cause of death: cardiogenic shock. Main disease: idiopathic dilated cardiomyopathy. (**Prof. Vera Demarchi Aiello, MD**)

## Comments

The patient was a marathoner with symptoms compatible with heart failure in the preceding year. He had syncope after physical training, being hospitalized. During his hospitalization, dilated cardiomyopathy was identified. The postmortem examination revealed moderate hypertrophy and dilatation of cardiac chambers. Clinically speaking, there was doubt regarding the etiology of the cardiomyopathy, and whether it would be related to the so-called "athlete's heart". Data from the literature have shown that, although some athletes, mainly practitioners of aerobic sports, can have dilated cardiac chambers, most have ventricular dimensions within the normal range. In around 10% of those athletes, the intensity of the dilatation is similar to that occurring in dilated cardiomyopathy, the differential diagnosis being established by the lack of systolic dysfunction, with maintenance of ventricular ejection fraction or even its increase. The same European authors have demonstrated that ventricular dilatation is positively related to the athlete's body surface and height.^8,9^ Therefore, although our patient was an athlete (marathoner) and had myocardial dilatation and hypertrophy, there was cardiac dysfunction, characterizing dilated cardiomyopathy. In addition, the microscopic exam evidenced marked pathological myocardial changes, such as cardiomyocyte hypertrophy and myocardial interstitial fibrosis, as well as organized or organizing microinfarcts.

Although infarcts were identified in the territories of the cerebral and splenic arteries, cardiac thrombi, a possible embolic source, were not evidenced. The postmortem exam showed no infection sign. Despite the report of rheumatic disease during childhood, his heart valves showed no lesion compatible with sequelae of that disease. (**Prof. Vera Demarchi Aiello, MD**)
